# The Interplay between Parental Monitoring and the Dopamine D4 Receptor Gene in Adolescent Cannabis Use

**DOI:** 10.1371/journal.pone.0049432

**Published:** 2012-11-28

**Authors:** Roy Otten, Edward D. Barker, Anja C. Huizink, Rutger C. M. E. Engels

**Affiliations:** 1 Behavioural Science Institute, Radboud University Nijmegen, Nijmegen, The Netherlands; 2 University of London, Birkbeck College, London, United Kingdom; 3 Developmental Psychology, VU University Amsterdam, Amsterdam, The Netherlands; Nathan Kline Institute for Psychiatric Research and New York School of Medicine, United States of America

## Abstract

**Background:**

Both environmental risk and genetic variation is believed to play a role in substance use. A candidate environmental variable is parenting. Recent studies have found support for the idea that the dopamine system affects the susceptibility to environmental influences. In the present study we will examine the interplay between effects of parental monitoring and the presence of the DRD4 7-repeat allele in adolescent lifetime cannabis use and the developmental course of cannabis use.

**Methods:**

A total of 311 adolescents participated in a five-wave longitudinal design. First, we conducted logistic regression analyses to examine the prospective associations between parental monitoring, the DRD4 polymorphism, their interaction and lifetime cannabis use. Second, individual growth parameters were calculated for frequency of cannabis use. Linear regression was used to assess the relationship between parental monitoring, the DRD4 polymorphism, their interaction, and the frequency of cannabis use.

**Results:**

There were no significant main effects of parental monitoring or the DRD4 polymorphism. However, both analyses showed that over a period of four years, a) when experiencing low levels of parental monitoring, individuals with the 7-repeat allele were more likely to show lifetime cannabis use and a stronger increase in frequency of cannabis use than individuals without this allele; b) when experiencing high levels of parental monitoring, individuals with the 7-repeat allele were less likely to show lifetime cannabis use and they showed a smaller increase in frequency of cannabis use than individuals without the 7-repeat allele.

**Conclusions:**

This study shows that carriers of the DRD4 7-repeat allele are disproportionally affected by the negative and positive effects of parental monitoring such that carriers of the DRD4 7-repeat allele, as compared to non-carriers, are more likely to use cannabis when levels of parental monitoring are low, and less likely to use cannabis when parental monitoring levels are high.

## Introduction

With a worldwide annual use prevalence of 2.5%, cannabis is the most cultivated, trafficked and abused illicit drugs [1]. The consequences of cannabis use over a prolonged period of time are serious and diverse, including heightened risks for impairment of cognitive functioning [2], depressive symptoms [3], and psychosis and schizophrenia [4]. Due to these striking impairments, research calls have highlighted the importance of identifying environmental and biological risks for initiation of cannabis use and more regular patterns of use. In the present study, we will concentrate on the role of parental monitoring (i.e., the extent to which adolescents perceive their parents to be controlling their whereabouts and activities) [5] in lifetime use and the developmental course of adolescent cannabis use. Studies have shown that children who report high levels of parental monitoring are insulated from engaging in risky behaviors, one of which is substance use [6], [7]. Through parental monitoring, parents can reduce the opportunity to engage in cannabis use [8]; they can create a milieu that attenuates adolescents' attitudes towards cannabis use; or they can limit the exposure to high-risk peers [9].

Recent approaches in psychopathology have stressed the interplay of environmental with genetic influences as mechanisms of risk [10]. In looking at gene-environment interactions, the *diathesis-stress model* has long been the paramount framework. In accordance with this model, some individuals are disproportionately likely to be affected by environmental stressors due to vulnerability in their genetic make-up [11], [12]. Another manner in which to frame a gene-environment research question is within the *differential susceptibility framework*, which postulates that individuals with particular genetic characteristics are disproportionally affected by negative *and* positive environments, such that susceptible genetic individuals can also “thrive” within highly supportive environments [13], [14].

In the present study we concentrate on the interaction between parental monitoring with a prominent gene from the dopaminergic system. Particularly, we will focus on a polymorphism which has a Variable Number of Tandem Repeats (VNTR) in exon 3 of the gene encoding the dopamine D4 receptor, which is represented by common length variants of 2, 4, or 7 repeats in most populations (i.e., the DRD4 7-repeat allele) [15]. Compared to other number of repeats, the DRD4 7-repeat allele alters the function of the encoded receptor by making it less sensitive to dopamine [16]. The DRD4-7 repeat allele has been associated with several behaviors and its attenuated response to dopamine produced by the 7-repeat allele is thought to associate with addiction and addiction-related phenotypes [17], [18]. Genetic factors have been identified as significant contributors to cannabis use with estimates of heritability ranging from 0.17 to 0.67 [19]. Nonetheless, with respect to cannabis use, direct effects of the DRD4-7 repeat allele have not yet been established [20].

The dopamine D4 receptor gene (DRD4) has also been primed as a prime candidate for gene-environment interplay. Specifically, the DRD4 has been targeted as a susceptibility gene in many studies on gene-environment interactions with a focus on parenting [13], [14]. For instance, one study showed that children (age 18–21 months) with the 7-repeat allele were influenced by parenting quality, while children without the 7-repeat allele were not [21]. In addition, Bakermans-Kranenburg and colleagues conducted a randomized controlled trial aimed at testing the genetic differences in explaining variability in the effects of an intervention that promoted positive parenting and sensitive discipline for 1 to 3 year olds (VIPP-SD) [22]. The effects of the program were strongest (i.e., most effective in decreasing externalizing behavior) for children carrying the DRD4 7-repeat allele. The authors argue that through this specific program, “parental sensitive responses to the children's signals and prompt reactions to disciplinary transgressions are stimulated, enhancing the reward value of the parent”. These findings suggest that DRD4 7-repeat allele children are more susceptible to parenting influences.

Whereas most studies looking at moderation of the effects of parenting by the DRD4 concentrated on young children, research concentrating on adolescents is scarce with mixed results. One recent study by Beach and colleagues [23] showed that youths (mean age 11.65) carrying the DRD4 7-repeat allele were more responsive to a parenting program than youths without this genotype. Specifically, they found support for the “differential susceptibility to parenting” hypothesis, illustrating greater preventive effects for youths carrying the DRD4 7-repeat allele, in the escalation of substance use in adolescence. We are aware of two other studies in which scholars looked at the interplay between genes related to the dopamine system and parenting in the developmental course of adolescent substance use. The first study concentrated on the direct effect of the DRD4 on regular cannabis use (on at least four occasions in the past four weeks) and alcohol use (on ten or more locations in the past four weeks), as well as the interaction with parenting (rejection, overprotection, and emotional warmth) [24]. No support was found for a direct genetic effect on cannabis or alcohol use. Nor was there support for an interaction between the DRD4 and parenting. In another study focusing on the interplay between the dopamine D2 receptor gene (another dopamine receptor subtype) and parenting in the developmental course of adolescent substance use, scholars found that adolescents with parents who were highly permissive towards alcohol consumption and carrying a genotype with the DRD2 A1 allele, used significantly more alcohol over time than adolescents without those characteristics [25].

### The present study

The objective of the present study was to test the role of parental monitoring, the DRD4 7-repeat allele and their interplay in lifetime use and the developmental course of cannabis use over a period of four years. In addition to a more traditional analytic approach we will use analyses that allow more optimal use of longitudinal data. We will control for important variables in cannabis use to ensure the integrity of the results. Specifically, cannabis use has been found related with tobacco use [26], lower socioeconomic status [27], and different aspects of personality [28]. Rather than a direct effect, we expect an interaction between parental monitoring and the DRD4 7-repeat allele. As compared to individuals without the DRD4 7-repeat allele, DRD4 7-repeat allele individuals may be disproportionally vulnerable for cannabis use under low levels of parental monitoring (accordingly with the diathesis-stress model). Alternatively, compared to individuals without the DRD4 7-repeat allele, DRD4 7-repeat allele individuals may be disproportionally affected by low *and* high levels of parental monitoring (in accordance with the differential susceptibility hypothesis). Particularly, in this case, individuals with the DRD4 7-repeat allele would be more at risk to use cannabis when parental monitoring is low, and less at risk to use cannabis when parental monitoring is high, as compared to non-carriers of the DRD4 7-repeat allele. Finally, although we look at parental monitoring (instead of rejection, overprotection and emotional warmth) and we use different outcome measures for cannabis use, it may be that the DRD4 has no effect on the relationship between parental monitoring and cannabis use, as was found by Creemers and colleagues [24].

## Methods

### Ethics Statement

The Central Committee on Research Involving Human Subjects in The Netherlands approved the protocols for the present study. We obtained written (parental) consent of all participants involved in the study.

### Participants

Participants were from the Family and Health Study, a prospective study among 428 families in the Netherlands that started in 2003 [29] (see Table 1 for descriptives).

**Table 1:** Descriptives.

Demographics	M (SD)	Percentage
Sex		53% Boys; 47% Girls
Age adolescent	15.22 (0.56)	
Age mother	43.82 (3.57)	
Age father; Country of birth	46.18 (4.00)	98.1% Netherlands; 1.9% Other
Education adolescent		30.2% Lower level education (i.e., preparatory secondary school for technical and vocational training); 29.3% Middle-level education (i.e., preparatory school for colleges below university level); 39.6% High-level education (i.e. preparatory secondary school for university)
Completed education father		1.4% Primary school; 17.9% Secondary school; 30.5% Technical and vocational school; 32.2% College; 17.4% University;
Completed education mother		2.1% Primary school; 31.4% Secondary school; 30.0% Technical and vocational school; 30.3% College; 5.4% University
Religion adolescent		54.7% Catholic; 22.1% Other; 23.2% No religion
Cannabis use for each time of assessment (Never use – tried but not during the last month – use during the last month)		Time 1: 90.0%–3.7%–6.3%
		Time 2: 80.7%–9.2%–10.2%
		Time 3: 73.3%–13.3%–13.3%
		Time 4: 64.0%–20.2%–15.7%
		Time 5: 60.2%–21.3%–18.5%

*Note*. Based on the total sample (n = 428).

Families were visited at home by interviewers. Questionnaires were filled out in private by each family member. We used data from five assessments with a one-year interval for the oldest child in the family. At year 1, participants for this study were between 14 and 17 years (*M* = 15.21, *SD*  = .60). The distribution of males and females was almost equal. More than 95% of the family members were of Dutch origin.

### Cannabis use

Information was collected using self-reports at each assessment point following two items: 1) have you ever used cannabis (0 =  Yes, 1 =  No); 2) How many times have you used cannabis during the last four weeks (1 =  Not, 2 = 1–2 times, 3 = 3–4 times, 4 = 5 times or more). We created a composite score, ranging from 0 to 2, which represented the frequency of cannabis use for each data collection wave (0 =  Never used, 1 =  Used, but not during the last four weeks, 2 =  One time or more during the last four weeks [26], [30].

### Parental monitoring

Maternal and paternal monitoring were both assessed with five items tapping the extent to which adolescents perceived their mothers and fathers to be controlling their whereabouts and activities (e.g., “Before I go out my mother tries to know what I will do and with whom I will spend time”). This questionnaire has been used frequently in prior research and its psychometric properties (including validity and reliability) have been proven to be good [31]–[33]. Response choices ranged from 1 (*never*) to 5 (*always*) with a high mean indicating higher maternal and paternal monitoring (*M*_maternal_ = 3.98, *SD*_maternal_  = 0.75; *M*_paternal_ = 3.47, *SD*_paternal_  = .99). Cronbach's alpha for both maternal monitoring and paternal monitoring were higher than. 75. Both variables were positively correlated (*r* = .63, *p*<.001) and combined to form an indicator for general parental monitoring.

### Genotyping

Saliva samples were collected for genetic analysis. A total of 311 adolescents could be genotyped after written informed consent by the parents and the adolescents. The 48-bp direct repeat polymorphism in DRD4 was genotyped as follows: From 10 ng genomic DNA a fragment was amplified in a 10 μ1 volume with 0.05 μM fluorescently labeled forward primer (Vic-5′-CGACTACGTGGTCTACTCG-3′) and reverse primer (5′-AGGACCCTCATGGCCTTG-3′), 0.4 mM dNTPs and 0.5 U La Taq (Takara, Lonza Verviers Sprl, Verviers, Belgium), in GC buffer (Takara, Lonza Verviers Sprl) with 1 M betaine. The cycling conditions for amplification involved 1 minute at 94°C, followed by 35 cycles of 30 seconds 94°C, 30 seconds at 58°C and 1 minute 72°C and an extra 5 minutes at 72°C. Subsequent determination of the length of the alleles was performed by direct analysis on an automated capillary sequencer (AB13730, Applied Biosystems, Nieuwerkerk a/d IJssel, The Netherlands) using standard conditions. Five percent duplicates and blanks were taken along as quality controls during genotyping. Hardy-Weinberg equilibrium (HWE) proportions were estimated from genotype information using the Markov-Chain Monte-Carlo approximation of the exact test implemented in the GENEPOP package V 3.3 [34]. No deviations from HWE were detected (*p* = .67). To maximize the power of the analyses, DRD4 genotypes were classified in two groups according to the absence or presence of the 7-repeat allele (respectively 65% versus 35%). Individuals with and without the 7-repeat allele did not differ on any of the adolescent characteristics that were used as covariates in this study, except for the personality dimension agreeableness. Children with the 7-repeat allele had higher scores on agreeableness (*OR*  = 2.05, 95% *CI*  = 1.29–3.27, *p*<.01).

### Covariates

#### Tobacco use

Participants were asked to indicate their smoking status on a nine-point ordinal scale [35]. We created a composite score ranging from 0 to 2 (0 =  Never used (54%), 1 =  Used, but not during the last four weeks (36%), 2 =  One time or more during the last four weeks (10%)).

#### Parental education

As a proxy for socioeconomic status we assessed both maternal and paternal education level [36]. Parents were asked to indicate their highest attained education level on an eight-point ordinal scale, with higher scores indicating higher education levels (*M*_father_  = 6.04, *SD*_father_  = 1.66, *M*_mother_  = 5.63, *SD*_mother_  = 1.57).

#### Personality

To assess the factors of the Five-Factor Model of Personality we used the Quick Big Five, a well-validated instrument [37], [38]. In a list consisting out of 30 traits, the respondent was asked to rate on a 7-point scale to what degree he/she possessed the concerned trait. Openness was measured with items such as creative, artistic and versatile (*α* = .70, *M* = 4.87, *SD*  = 0.84); conscientiousness was measured with items such as organized, orderly and efficient (*α* = .85, *M* = 4.19, *SD*  = 1.14); extraversion was measured with items such as quiet, withdrawn and shy (*α* = .84, *M* = 4.78, *SD*  = 1.09); agreeableness with items such as kind, likeable and cooperative (*α* = .77, *M* = 5.45, *SD*  = 0.64); and emotional stability was assessed with items such as nervous, fearful and sensitive (*α* = .73, *M* = 4.29, *SD*  = 0.93).

#### Attrition analyses

Adolescents who were genotyped did not differ from those who were not genotyped on any of the study variables. With respect to the outcome variable, of the dataset including all participants who were genotyped and thus included in our study, data on cannabis use were available for all participants at T1, 98.4% of the cannabis use data were available at T2; 94.6% at T3; 87.6% at T4 and 83.4% of the data were available at T5. For the first set of analyses we only use data from time 1 and time 5. However, for the second set of analyses, we also used data obtained from the other time points. To make use of all available data, genotyped participants with at least one data point on cannabis use were allowed in the latent growth curve analyses. Participants who had missings on cannabis use data on one of the five measurement points (26.1% of the participants) were not different on any of the study variables from those respondents for whom complete data over all time five points were available.

#### Statistical analyses

Two sets of analyses were conducted. The aim of the first set of analyses was to predict lifetime use of cannabis. To do this, we selected all never users. By means of multivariate logistic regression we predicted any experience with cannabis use four years later (i.e., the dependent variable), by parental monitoring, the DRD4 genotype, and covariates (i.e., age, sex, smoking, SES, and personality) (i.e., the independent variables). In a second block, we included an interaction term to test whether the effects of parental monitoring on cannabis use onset would be different for individuals with and without the DRD4 7-repeat allele (parental monitoring*DRD4).

In the second set of analyses, latent growth curves were estimated to look at development of frequency of cannabis use over the five waves. Latent growth curve modeling permits to capture the initial levels of individuals at the beginning of a developmental period, and individual changes over the developmental period and, thus, individual developmental pathways [39]. Two individual growth-curve parameters (i.e., factor scores) were retained for subsequent analyses: the intercept (i.e., initial level of cannabis use) and the slope (i.e., rate of growth in cannabis use). In a second step we used linear regression analyses to predict the growth curve parameters (i.e., intercepts and slopes) of cannabis use consequently, one by one, by parental monitoring, the DRD4, and we tested whether the effects of parental monitoring on the intercept and slope of cannabis use were different for adolescents with and without the 7-repeat allele of the DRD4 by including an interaction term (i.e., parental monitoring*DRD4), while controlling for potential confounders age, sex, smoking, SES, and personality. When predicting the slope we also controlled for the initial level of use (i.e., intercept). Analyses were based on data of adolescents who had data on cannabis use, parental monitoring, and the DRD4 (N = 300).

## Results

### Descriptives

Lifetime cannabis use increased from 10% at wave 1 to 39.8% at wave 5, while monthly use increased from 6.4% at wave 1 to 18.3% at wave 5. In table 2, we correlated a mean score of lifetime use of cannabis over the five measurements with all the study variables. The mean score of lifetime use of cannabis was only marginally related with parental monitoring (*p* = .09), and sex (*p* = .06). Individuals who reported lifetime use were more open (*p*<.01), extravert (*p*<.01), less conscientious (*p*<.01), and more likely to smoke (*p*<.01). Presence of the DRD4 7-repeat allele was associated with higher levels of parental monitoring (*p*<.05) and higher levels of agreeableness (*p*<.01).

**Table 2:** Bivariate correlations among study variables.

	1	2	3	4	5	6	7	8	9	10	11	12
1. Cannabis use	-											
2. DRD4 7-repeat allele	−.04	-										
3. Age	.05	−.03	-									
4. Sex	−.11†	−.02	.01	-								
5. Parental monitoring	−.10†	.14*	−.03	.14*	-							
6. Education father	−.06	.01	−.02	−.04	.08	-						
7. Education mother	.10	.03	.04	.03	.08	.43**	-					
8. Smoking	.52**	−.08	.11†	.02-	−.14*	−.17**	−.05	-				
9. Openness	.12*	−.01	.07	−.08	.15**	−.03	.00	.05	-			
10. Conscientiousness	−.22*	.02	−.01	.12*	.15*	−.06	−.08	−.11†	.12*	-		
11. Extraversion	.18*	.01	−.09	−.03	.01	−.04	.03	.22**	.19**	−.15*	-	
12. Agreeableness	−.03	.16**	.00	.01	.17**	.08	−.02	.00	.39**	.30**	.19**	-
13. Emotional stability	.06	.00	−.05	−.18**	−.08	.02	−.00	.03	−.09	−.11	.36**	.04

*Note*. The numbers in the top row of the table correspond to the variables in the first column. DRD4 7-repeat allele is code 0 = 7 repeat allele absent, 1 = 7-repeat allele present. *  =  *p*<.05, **  =  *p*<.01, two-tailed tests. †  =  marginally significant.

#### Multivariate logistic regression

Table 3 shows the results of the logistic regression. In the first block we included parental monitoring and the DRD4 as well as the covariates. In the second block we entered the interaction term (parental monitoring*DRD4). Being male, smoking, and higher levels of extraversion were associated with higher odds for lifetime cannabis use. There were no significant effects of parental monitoring or the DRD4. However, there was a strong and consistent interaction between parental monitoring and the DRD4.

**Table 3:** Multivariate logistic regressions of DRD4 and parental monitoring on lifetime use of cannabis.

Block	Predictors	Initial model		Education level		Smoking		Personality	
		OR	CI	OR	CI	OR	CI	OR	CI
Block 1	DRD4	.94	.52–1.69	.93	.51–1.69	.96	.52–1.80	.97	.52–1.81
	Age	.68	.42–1.09	.65	.40–1.06	.66	.40–1.08	.69	.42–1.12
	Sex	.56*	.32–1.00	.57	.32–1.02	.54*	.30–.98	.56	.31–1.02
	Parental monitoring	.88	.60–1.29	.85	.58–1.25	.95	.64–1.42	.89	.60–1.33
	Father's education			1.18	.97–1.45				
	Mother's education			.97	.80–1.17				
	Smoking					2.78***	1.73–4.47		
	Openness							1.25	.84–1.86
	Conscientiousness							.76	.58–1.01
	Extraversion							1.34*	1.00–1.81
	Agreeableness							.94	.56–1.58
	Neuroticism							.93	.67–1.29
Block 2	DRD4*Parental monitoring	.29**	.12–.68	.30**	.12–.69	.27***	.11–.67	.27**	.11–.66

*Note*. OR  =  Odds Ratio (lifetime cannabis use is dependent variable), CI  = 95% Confidence Interval. *  =  *p*<.05, **  = *p*<.01, ***  =  p<.001, two-tailed tests.

To scrutinize the nature of the interaction, we conducted a median split on parental monitoring to distinguish between high and low levels of parental monitoring in our sample and we plotted the interaction.

**Figure 1:**
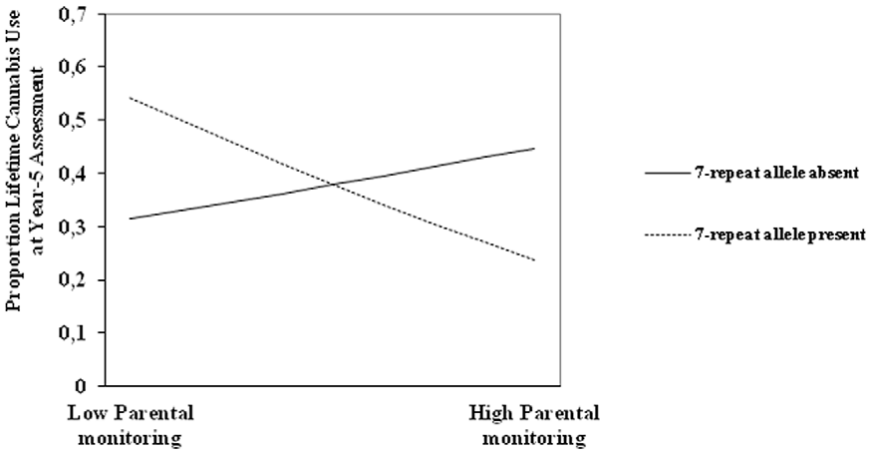
Moderation of the DRD4 7-repeat allele on the link between parental monitoring and lifetime use of cannabis over four years.

**Figure 2:**
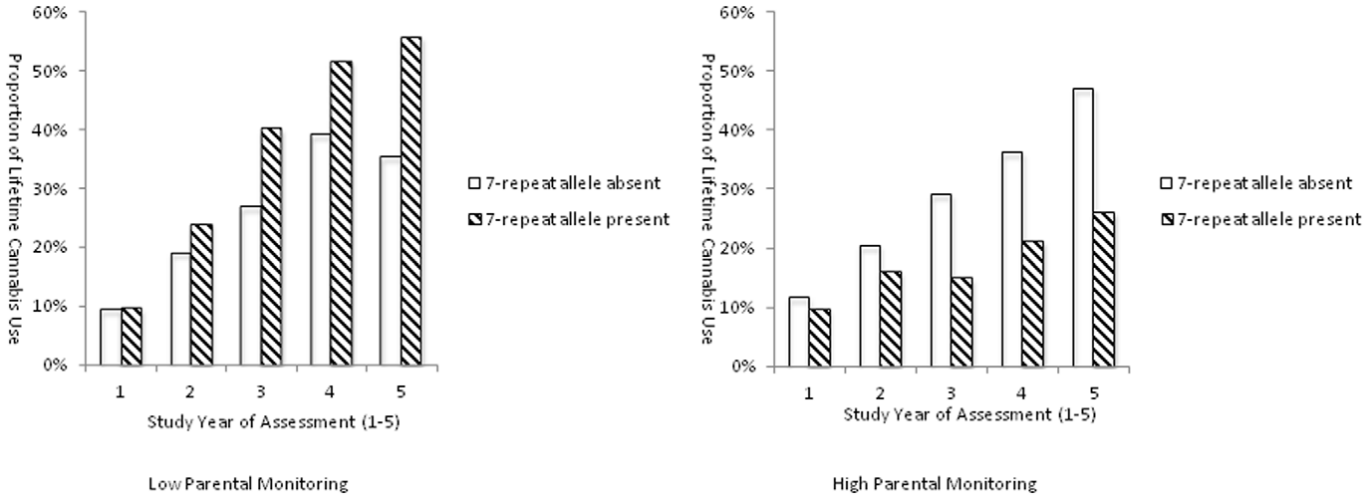
Proportion of lifetime users of cannabis at study year of assessment, separated for carriers and non-carriers of the DRD4 7-repeat allele, under conditions of low versus high levels of parental monitoring.

Figure 1 shows that under low levels of parental monitoring, individuals with the DRD4 7-repeat allele were more likely to report lifetime cannabis use than individuals without the DRD4 7-repeat allele. When levels of parental monitoring were high, individuals with the DRD4 7-repeat allele were less likely to report lifetime use than individuals without the 7-repeat allele. We also looked at differences in proportions of adolescents reporting lifetime cannabis use in individuals with and without the DRD4 7-repeat allele under conditions of high and low levels of parental monitoring at each of the five time points (Figure 2), illustrating the same pattern.

#### Latent growth curves

Table 4 gives the fit indices, mean initial values (i.e., intercepts), and mean linear rates of changes (i.e., slopes) as well as the variability in initial levels and linear rates of change. The relative fit indices for cannabis use were satisfactory.

**Table 4:** Model fit Indices for growth curve parameters.

	*X* ^2^(*df*)	p	*CFI*	*TLI*	*RMSEA*	*Mean Intercept*	*Mean Slope*	*Variance Intercept*	*Variance Slope*
Cannabis use	13.871 (6)	0.03	0.98	0.97	0.07	1.17	0.11	0.47	0.04
						SE = 0.03	SE = 0.01	SE = 0.08	SE = 0.01
						(38.21)***	(9.92)***	(6.03)***	(4.72)***

*Note*. X^2^(df)  =  Robust chi-square with estimated degrees of freedom; CFI  =  Comparative Fit Index [47]; TLI  =  Tucker-Lewis Index [48]; RMSEA  =  Root Mean Squared Error of Approximation [49]. T-values are presented in parentheses below their respective associated growth curve parameter. *p*<.001, two-tailed tests.

In a second step we predicted the growth parameters of cannabis use by means of eight linear regression analyses (Table 5). For instance, column 5 depicts estimates resulting from a regression analysis predicting the intercept of cannabis use while controlling for the DRD4, age, sex, parental monitoring, and father's and mother's education. In column 6 estimates are shown from a regression analysis predicting the slope of cannabis use, while controlling for the same covariates as in column 5 plus the intercept of cannabis use.

**Table 5:** Linear regression of DRD4 and paternal and maternal monitoring on intercept and slope of cannabis use controlling for covariates.

Block	Predictors	Initial model		Education level		Smoking		Personality	
		I	S	I	S	I	S	I	S
Block 1	Intercept		−.70***		−.71***		−.78***		−.72***
	DRD4	.03	−.02	.03	−.02	.05	−.00	.04	−.02
	Age	.20***	−.05	.20***	−.07	.17**	−.06	.20***	−.05
	Sex	.05	−.15***	.03	−.14***	.04	−.15***	.06	−.12***
	Parental monitoring	−.08	−.03	−.09	−.03	−.04	−.01	−.09	−.04
	Father's education			−.15*	−.01				
	Mother's education			.12	.11*				
	Smoking					.36***	.22**		
	Openness							.09	.09*
	Conscientiousness							−.08	−.11**
	Extraversion							.07	.07
	Agreeableness							−.03	.03
	Neuroticism							−.02	.01
Block 2	DRD4*Parental monitoring	−.07	−.15**	−.05	−.15*	−.07	−.15**	−.09	−.14**

*Note*. I  =  Intercept, S  =  Slope. I and S are the dependent variables in the eight separate linear regression models.

* =  *p*<.05, **  =  *p*<.01, ***  =  p<.001, two-tailed tests.

In the first block the DRD4 and parental monitoring were included in the model to predict the initial level of cannabis use (I) or change over time (S), while controlling for covariates. In the second block we entered the interaction term (parental monitoring*DRD4). Age and smoking were positively and education of father was negatively related to the intercept of cannabis use (columns 2, 4, 6, 8). There were no significant main effects of parental monitoring or the DRD4 genotype on the intercept of cannabis use and there was no support for moderation.

**Figure 3:**
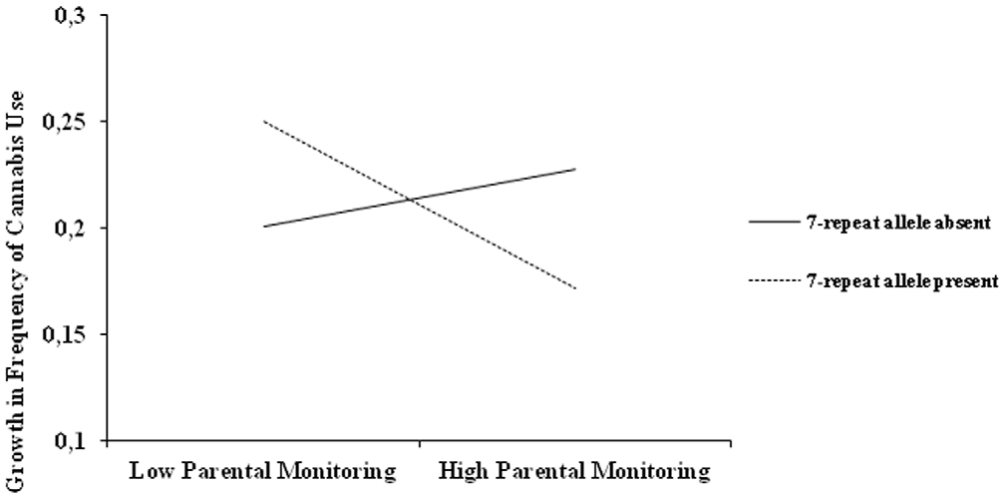
Moderation of the DRD4 7-repeat allele on the link between parental monitoring and growth in frequency of cannabis use.

When predicting the slope we controlled for initial levels of cannabis use (columns 3, 5, 7, 9). Adolescents with higher initial levels of use had lower growth rates than adolescents with lower initial levels of use. Moreover, adolescents with mothers with a higher level of education; adolescents who smoked; and adolescents with a more open personality were more likely to progress to more frequent levels of use. In contrast, girls and adolescents with higher conscientiousness scores were less likely to progress to more frequent levels of use. There were no significant main effects of parental monitoring or the DRD4 genotype on the slope of cannabis use; however, we did find a consistently significant interaction effect of the DRD4 on the link between parental monitoring and the growth of cannabis use. Controlling for all covariates this interaction effect remained significant. A multigroup analysis showed that the effect of parental monitoring on the development of cannabis use was only significant in the presence of the DRD4 7-repeat allele (*Β* = −.28, *p*<.01), and not in absence of the allele (*Β* = .05, *p* = .50). A Satorra-Bentler Chi-Square Difference test [40] showed that the effects were indeed significantly different for children with and without the DRD4 7-repeat allele (*p*<.01). Figure 3 shows that individuals with the DRD4 7-allele are disproportionally affected by low and high levels of parental monitoring in such a manner that under conditions of low parental monitoring DRD4 7-repeat allele individuals would be more likely to show a stronger increase in frequency of cannabis use over time than individuals without this genotype. Under conditions of high parental monitoring, DRD4 7-repeat allele individuals would be more likely to show a weaker increase in frequency of cannabis use over time than individuals without this genotype.

## Discussion

The objective of the present study was to test the direct effects of parental monitoring, the DRD4 7-repeat allele and their interplay on adolescent cannabis use. Although the presence of the DRD4 7-repeat allele was associated with higher levels of parental monitoring, direct effects on lifetime use or the developmental course of cannabis use (both the intercept and the slope) were not identified for either parental monitoring (parental monitoring and cannabis use were marginally related) or the DRD4 7-repeat allele. However, results *did* show that when experiencing (relatively) low levels of parental monitoring, adolescents with the DRD4 7-repeat allele were more likely to show lifetime use of cannabis, as well as accelerated progression to more frequent patterns of cannabis use as compared to adolescents without the DRD4 7-repeat allele. In contrast, when experiencing (relatively) high levels of parental monitoring, adolescents with the DRD4 7-repeat allele were less likely to show lifetime use of cannabis as well as slower progression towards frequent patterns of use than adolescents without the DRD4 7-repeat allele. These results are in line with other studies showing that the presence of the DRD4 7-repeat allele heightens susceptibility to supportive/positive as well as unsupportive/negative environmental influences [13], [14], [21]–[23].

Studies have suggested that cannabinoids (the primary psychoactive ingredient of cannabis) may indirectly activate mesolimbic dopaminergic pathways, making dopaminergic receptor genes important candidate genes for cannabis use. Although associations have been found between the DRD2 and cannabis use [20], there are no studies linking the DRD4 directly to cannabis use, so our results are in line with the literature. This adds to the idea that the DRD4 should be looked at as a gene that modifies the susceptibility to environmental influences. There was also no direct effect of parental monitoring on cannabis use, which is not in line with the literature [8]. It may be that, in general, parents in our sample scored high on parental monitoring, which made it more difficult to detect a direct effect of parental monitoring.

The present study extends knowledge of previous studies in a few ways. First, while the number of studies concentrating on gene-environment interactions rapidly increases [10], this is the first study to illustrate the interplay between genes and environment in cannabis use in a longitudinal design. More research is needed to understand whether results can be replicated also with respect to other forms of adolescent substance use. Second, in addition to a more traditional statistical approach (i.e., logistic regression analysis), the present study looked at the developmental course of cannabis use, showing that a gene-environmental interaction can indeed be detected when making efficient use of repeated measures by assessing the rates of change. Our results are not in line with findings of Creemers and colleagues who did not find support for a gene-environment interaction [24]. However, this study differed substantially: as we looked at parental monitoring, Creemers and colleagues concentrated on rejection, overprotection, and emotional warmth. Moreover, whereas we concentrated on lifetime use and changes in frequency of use over time by means of latent growth curves, the other study focused on regular use of cannabis (on at least four occasions in the past four weeks) and alcohol use (on ten or more locations in the past four weeks) by means of logistic regression.

Third, although the sample size in this study was small and findings need to be replicated, the present study shows support for the idea that so-called plasticity genes may make people more sensitive to their environment [13], [14], which has been found repeatedly in children but rarely in adolescents. The results fit within the differential susceptibility framework. Although this framework offers some interesting hypotheses, more research is needed to test this framework and its theoretical underpinnings (i.e., the basic idea of differential susceptibility stems from the evolutionary argument that children should vary in their susceptibility to parental rearing to optimalize reproductive fitness of offspring). Furthermore, more studies are needed to better understand the underlying biological mechanisms making individuals with the DRD4 7-repeat allele (or other polymorphisms) more responsive to parenting. In addition, research should focus on delineating the specific environmental characteristics that trigger these biological processes. Whereas the DRD4 has been referred to as the ‘parenting’ gene, it should be specified what specific aspects of parenting ‘trigger’ differential susceptibility. Finally, it should be tested whether differential susceptibility is limited to parenting or whether a similar phenomenon could be detected when looking at the supportive and aversive aspects of peer influence.

Whereas this study concentrated on the interplay between genes and environment, we cannot rule out the possibility of a gene-environment correlation [41]. Specifically, as parental monitoring was correlated with the DRD4 (i.e., presence of the 7-repeat allele), it is possible that the individuals' genetically influenced behavior or personality evoked parental monitoring (e.g., evocative gene-environment correlation).

### Limitations

Some limitations of this study should be mentioned. A primary shortcoming of this study was that we did not include a positive outcome, parallel in measurement to cannabis use, so true differential susceptibility could not be tested (i.e., the test for specificity by replacing susceptibility factors) [13]. For example, recent research [42] has shown that impulsive adolescents with parents who monitor their activities and whereabouts are more likely to engage in prosocial and positive community activities. Second, all included variables on cannabis use were assessed with self-reported frequency. Self-report with respect to cannabis may be prone to error. However, measurement of cannabis use by physiological measures is difficult due to variation in biologically available cannabinoids concentrations. Therefore, a combination of both self-report and more objective measures [43] would have provided more valid measures. A third limitation refers to the sample and sample size, which was mentioned before. We are aware that GxE findings remain controversial and that there is a lot of concern about publication biases, problems with statistical power, and high false discovery rates, as a consequence of small sample sizes [44], [45]. In fact, we computed the achieved power for the present paper (post-hoc) for both sets of analyses that we conducted (i.e., logistic regression on lifetime use and linear regression on frequency of use). The power that we achieved (with 0.05 error probability) with logistic regression on lifetime use (interaction effect size R^2^ = .053) was good (.99). With respect to linear regression (interaction effect size R^2^ = .013) power was low (.60). Nevertheless, our findings are relatively robust as they could be illustrated by means of different analyses and different outcome variables. However, we emphasize the importance of replication with larger samples. Fourth, our sample included only intact families with both fathers and mothers. This inclusion criterion may have lead to an underestimation of cannabis use in this specific age group, as it has been shown that cannabis use is higher in children who grow up in single-parent families [46]. Moreover, levels of parental monitoring in the participating families were generally high. It is likely that the inclusion of more diverse families would have lead to a larger range of parental monitoring.

### Conclusion

In sum, this is the first gene-environment study providing evidence for interplay between parental monitoring and the DRD4 genotype affecting lifetime use and the developmental course of adolescent cannabis use. Specifically, compared to non-carriers of the DRD4 7-repeat allele, carriers were more at risk for cannabis use when parental monitoring was low and less at risk when parental monitoring was high. These findings fit within the differential susceptibility framework.
